# Mitigating the Psychological Impact of COVID-19 on Healthcare Workers: A Digital Learning Package

**DOI:** 10.3390/ijerph17092997

**Published:** 2020-04-26

**Authors:** Holly Blake, Fiona Bermingham, Graham Johnson, Andrew Tabner

**Affiliations:** 1 School of Health Sciences, University of Nottingham, Nottingham NG7 2HA, UK; 2 National Institute for Health Research (NIHR) Nottingham Biomedical Research Centre, Queen’s Medical Centre, Nottingham NG7 2UH, UK; 3 Leicester Medical School, University of Leicester, Lancaster Rd, Leicester LE1 7HA, UK; feb7@student.le.ac.uk; 4 REMEDI Emergency Department, Royal Derby Hospital, Uttoxeter Rd, Derby DE22 3NE, UK; graham.johnson4@nhs.net (G.J.); andrew.tabner@nhs.net (A.T.)

**Keywords:** COVID-19, coronavirus, pandemic, wellbeing, mental health, digital, e-learning

## Abstract

The coronavirus pandemic (COVID-19) will undoubtedly have psychological impacts for healthcare workers, which could be sustained; frontline workers will be particularly at risk. Actions are needed to mitigate the impacts of COVID-19 on mental health by protecting and promoting the psychological wellbeing of healthcare workers during and after the outbreak. We developed and evaluated a digital learning package using Agile methodology within the first three weeks of UK outbreak. This e-package includes evidence-based guidance, support and signposting relating to psychological wellbeing for all UK healthcare employees. A three-step rapid development process included public involvement activities (PPIs) (STEP 1), content and technical development with iterative peer review (STEP 2), and delivery and evaluation (STEP 3). The package outlines the actions that team leaders can take to provide psychologically safe spaces for staff, together with guidance on communication and reducing social stigma, peer and family support, signposting others through psychological first aid (PFA), self-care strategies (e.g., rest, work breaks, sleep, shift work, fatigue, healthy lifestyle behaviours), and managing emotions (e.g., moral injury, coping, guilt, grief, fear, anxiety, depression, preventing burnout and psychological trauma). The e-package includes advice from experts in mental wellbeing as well as those with direct pandemic experiences from the frontline, as well as signposting to public mental health guidance. Rapid delivery in STEP 3 was achieved via direct emails through professional networks and social media. Evaluation included assessment of fidelity and implementation qualities. Essential content was identified through PPIs (*n* = 97) and peer review (*n* = 10) in STEPS 1 and 2. The most important messages to convey were deemed to be normalisation of psychological responses during a crisis, and encouragement of self-care and help-seeking behaviour. Within 7 days of completion, the package had been accessed 17,633 times, and healthcare providers had confirmed immediate adoption within their health and wellbeing provisions. Evaluation (STEP 3, *n* = 55) indicated high user satisfaction with content, usability and utility. Assessment of implementation qualities indicated that the package was perceived to be usable, practical, low cost and low burden. Our digital support package on ‘psychological wellbeing for healthcare workers’ is free to use, has been positively evaluated and was highly accessed within one week of release. It is available here: Supplementary Materials. This package was deemed to be appropriate, meaningful and useful for the needs of UK healthcare workers. We recommend provision of this e-package to healthcare workers alongside wider strategies to support their psychological wellbeing during and after the COVID-19 pandemic.

## 1. Introduction

In January 2020, the World Health Organisation (WHO) declared the outbreak of a new coronavirus disease (COVID-19) to be a Public Health Emergency of International Concern. In March 2020, the WHO made the assessment that COVID-19 would be characterised as a pandemic. Protecting the mental wellbeing of healthcare workers caring for people with COVID-19 has been identified as imperative for the long-term capacity of the health workforce [1]. In particular, providing psychological support to frontline workers is noted to be a significant public mental health challenge over the coming weeks and months [2].

There is a clear need for immediate action to safeguard the welfare of the health and care workforce [3]. In addition to fears around COVID-19 exposure, anxieties related to shortages of personal protective equipment (PPE) or other essential equipment and the challenges of family support and childcare while they work, healthcare workers may experience irregular hours and higher workloads, coupled with anxiety, as they enter new or unfamiliar clinical roles [4,5]. They are at risk of emotional strain and physical exhaustion from the provision of care to growing numbers of patients who may then rapidly deteriorate; they may be exposed to critical illness or death of their co-workers [4] and they may also face moral dilemmas in decision making around provision of care with limited resources [6].

Stress, anxiety and depression may be viewed as normal emotional reactions in the face of a pandemic [7]. Healthcare workers in previous pandemics have experienced high levels of stress, anxiety and low mood ([8]: A/H1N1 influenza; [9,10]: severe acute respiratory syndrome, SARS), with negative psychological impacts sustained after one year ([10]: SARS). Symptoms of post-traumatic stress (PTSD) have been observed within weeks of an outbreak ([9]: SARS).

The psychological impacts on employees have negative consequences for organisations. The extreme pressures experienced by healthcare workers during a pandemic may increase their risk of burnout, which has adverse outcomes not only for individual wellbeing, but also for patient care and the healthcare system [11]. During the SARS outbreak, emotions experienced by healthcare workers were associated with resignations and poor work performance [12].

Healthcare workers at higher risk of exposure to the virus experience a greater psychological impact than those with less exposure (e.g., [10]: SARS). In China, frontline healthcare workers caring directly for patients with COVID-19 experienced stress, anxiety and insomnia, and exhibited higher levels of severe mental health symptoms than those in secondary roles [13,14,15]. Conversely, other studies have shown a higher prevalence of psychological distress among non-frontline staff, possibly due to these workers having less access to information and psychological support [16]. This highlights that support mechanisms are needed for all healthcare workers, irrespective of their job role or level of virus exposure.

Psychological support should focus on organisational as well as individual characteristics, with ‘a broader goal of maintaining an organisational culture of resilience’ [17]. Prior pandemics have demonstrated that the context of the organisation has powerful effects on psychological outcomes for the workforce ([9]: SARS). It is well established that cultural norms within an organisation, leadership styles, and patterns of management communication are known to be key factors in worker stress [18]. In pandemic situations, clear communication of directives and precautionary measures reduces the likelihood of emotional distress, as does peer support ([9]: SARS). Social support outside of the workplace may also buffer stress, but healthcare workers often neglect relationships with their friends and family due to heavy workloads or concerns around infecting others due to their own occupational exposure to the virus. Maintenance of social contact is increasingly challenging in the context of social distancing requirements and, anecdotally, there are reports of healthcare workers experiencing social stigma and abuse due to public fears of contracting the virus from those with greatest exposure.

The additional uncertainty around COVID-19 progression and treatments as well as the challenges of limited resources means that healthcare workers will certainly face difficult decisions and moral dilemmas during the pandemic. This can result in moral injury, described as ‘the psychological distress which results from actions, or lack of them, which violate someone’s moral or ethical code’ [19]. There are UK media reports of the benefits of psychological first aid (PFA) for healthcare workers to support individual coping skills and resilience during the COVID-19 crisis (e.g., [5] RTÉ Ireland News Report: 9 April 2020). Greater promotion of self-care is needed (e.g., healthy eating, hydration, physical activity) since, during a pandemic (as well as at other times), healthcare workers often deprioritise their own health and wellbeing in favour of patient care [9].

Research has identified that healthcare professionals have requested five things from their employer during the COVID-19 pandemic: hear me, protect me, prepare me, support me, and care for me [20]. For the healthcare workforce to perform to their full potential over an extended time period, healthcare employers must provide early psychosocial support for all employees that addresses these requests and is focused on: creation of a psychologically safe environment, strong leadership, clear organisational strategies for staff wellbeing, consistent communication and significant team support. Such an environment will foster individual resilience and sanction self-compassion and self-care. Building a culture of organisational resilience may help to reduce the likelihood or severity of psychological manifestations requiring treatment once the immediate threat of COVID-19 subsides.

Online learning is being used successfully to provide training related to COVID-19 for policy makers and health and care workers, as well as the general public (e.g., the WHO, the National Health Service (NHS) Health Education England, FutureLearn). Digital approaches are also being used to educate future healthcare workers through Massive Open Online Courses (MOOC) (e.g., [21]: interns; [22]: medical residents). However, to our knowledge, in March 2020, there was no online learning package available that focused specifically on supporting the psychological wellbeing of healthcare workers. Our public engagement activities (described in the methods) indicated that the emerging COVID-19 information and advice related to psychological wellbeing was overwhelming, and widely dispersed, contributing to the ‘information overload’, a phenomenon often reported in the media [23].

The aim of this study was to synthesise evidence-based information to rapidly develop and evaluate a digital learning package to support psychological wellbeing for all healthcare workers. Content development was based on a series of public engagement activities, and the evaluation replicated pre-tested methods used in [24]. Evaluation included assessment of toolkit fidelity and implementation qualities to determine the acceptability, usability and utility of the package with healthcare workers.

## 2. Materials and Methods

This study reports on the development and evaluation of a digital learning resource and was, therefore, exempt from approval by an ethics committee. The authors adhered to the British Psychological Society (BPS) Code of Human Research Ethics (see https://www.bps.org.uk/news-and-policy/bps-code-human-research-ethics-2nd-edition-2014). The overall aim of the proposed study was to: (i) rapidly develop and evaluate a digital learning package to assist healthcare employers who are developing provisions for psychological wellbeing of healthcare workers during the COVID-19 pandemic; (ii) enable users to be better informed about psychological issues and impacts during and after a pandemic; (iii) normalise psychological responses to COVID-19 in healthcare workers; (iv) encourage help-seeking behaviour by providing evidence-based information, support and signposting for users.

This study was based on a three-step process, including public involvement activities, content and technical development with iterative peer review, delivery (number of users accessing the package plus Twitter engagement within 7 days of launch) and evaluation. Each step is reported separately as a distinct element of the study, combining methods and results.

The method was adapted from an approach used previously to develop and test digital resources for use in the workplace setting [24]. STEPS 1 and 2, through PPI work and iterative peer review, established the need for package development, and determined the broad areas of content to be included following an Agile approach. STEP 2 also involved the package content and technical development to establish the final, online version. STEP 3 demonstrated the extent to which the learning package could be delivered as intended and established the quality and utility of the package by the target audience.

Given the real-world focus of this study, the rapidity of COVID-19 planning, and the need for a timely product outcome, we adopted a pragmatic approach engaging stakeholders from healthcare and academia through the development process. We outlined timelines for development at the outset informed by PPI activities taking place in February and March 2020, with the package development and review being undertaken concurrently in a two-week period in March 2020. Evaluation was then undertaken within one week of public release of the package in early April 2020. The whole development, review and evaluation process for Version 1.0, therefore, took three weeks. The package was conceived by H.B. and developed by H.B. and F.B. We were guided by Agile science approaches utilising Kanban methodology (www.atlassian.com/agile/kanban) as described in our prior work [24]. This is an iterative process of development that is resource efficient and allows for continuous review and delivery. An outline of content is shown in Table 1, and significant ‘statements’ (direct quotations from other authors in the field) were inserted throughout the tool to enhance pertinent learning points.

We used co-design strategies and user experience testing, informed by stepwise processes used in the development of digital behaviour change interventions. These included:

- Pre-define; STEP 1: PPI activities to establish the need and understand the context.
- Define; STEP 1 and STEP 2: stakeholder consultation and peer review activities to define the package content.
- Design; STEP 2: Draft content and technical development by project team, with user testing conducted by the authors.
- Develop; STEP 3: Expert reviews leading to package refinement and production.
- Deploy; STAGE 4: Real-world fidelity testing with healthcare workers and healthcare students.

For expediency and rapid development, we modelled the process utilised by Blake and colleagues [24] with evaluation components aligned with established guidelines on process evaluation for public health interventions and research [25], and mapping of Research Questions (RQs) to the intervention as described by Murray and colleagues [26]; see Table 2. The relevant key components measured here included context (STEP 1 and Table 2), dose delivered, dose received, fidelity, and implementation.

## 3. Methods and Results

### 3.1. STEP 1: Stakeholder Consultation

Objectives: To determine the views of healthcare workers towards a digital resource to support psychological wellbeing at work, and to determine participant’s views of the package content and suggestions for change.

Methods: We held three stakeholder consultation groups between January and March 2020 and in March 2020 consulted with a further five experts who held strategic roles related to COVID-19 Employee Health and Wellbeing planning (PPI total: *n* = 97). Group 1 was undertaken in January (healthcare students, *n* = 35), Group 2 in February (Group 2: registered nurses, *n* = 25), and Group 3 in March (healthcare workers from nursing and the allied health professions, *n* = 32). The sessions were all 2 h in length and delivered by the lead author (H.B.), including a slide presentation on workplace health and wellbeing, followed by group discussion focused around workshop activities relating to psychological wellbeing for healthcare workers. Each group produced notes on their discussions using flip-chart paper, which were then presented back to the wider group by a self-nominated group ‘spokesperson’. The flip-chart papers with the group summary notes were then provided to the session lead. The two activities included discussion on:

Activity 1: Perceptions towards digital platforms for promoting health and wellbeing.
Activity 2: Key issues around psychological wellbeing for healthcare workers.

Additionally, individual telephone discussions were held with the 5 strategic role-holder PPI participants (3 nurses, 1 physiotherapist, 1 medical doctor) who provided further comment and suggestion around elements of the package content relating directly to COVID-19 and psychological wellbeing. With consent from the stakeholders, key points from the discussions were noted by the lead author, who then read these back to the proposer for confirmation of accuracy. For the purpose of this paper, all session attendees and the individual healthcare professionals are referred to collectively as stakeholders.

Results: Stakeholders were overwhelmingly positive about the use of digital technologies to promote health and wellbeing, due to the flexibility offered. It was raised that online materials needed to go beyond the generic promotion of health and must address issues that were specifically relevant to healthcare workplace environments (e.g., shift work), as well as specific issues experienced by healthcare workers during COVID-19 (e.g., dealing with difficult decisions and coping with guilt during self-isolation). Stakeholders generally agreed that content needed to be interactive and engaging, including links to external reports or guidance, signposting to interactive materials such as apps, and embedding video material. Quiz or wellbeing self-assessments were not deemed appropriate for this audience in pandemic circumstances—healthcare workers indicate that they preferred to know the information was collated and available, rather than being tested on their learning during stressful times. It was proposed by healthcare professionals that health and wellbeing training should be mandatory for all healthcare workers or, at minimum, widely promoted, and that a digital package would assist with wide circulation and adoption of the relevant material across healthcare settings, and geographical regions. Both healthcare students and registered healthcare professionals mentioned that providing materials to support psychological wellbeing, alongside other support mechanisms, would demonstrate that their employer (or university) valued them as individuals. All stakeholders expressed a preference for materials that were flexible to use, for example, a ‘dip-in, dip-out’ approach was seen to be more attractive than materials presented in modular format that had to be completed start to finish in a single sitting, or in a set order. A minority of the stakeholders expressed concerns regarding personal lack of technical skills and where to access technical support if required—it was, therefore, proposed that the package be developed in a free-to-access and simple format that did not require logging into a system or any specific technical expertise. Regarding content, some specific suggestions were made for inclusion of information on moral injury, decision making and anxiety, together with links to self-care resources such as free mindfulness apps, particularly those advocated by the UK National Health Service (NHS). It was deemed important to advocate the normalisation of psychological responses to the pandemic as a key message and highlight the importance of psychologically safe environments as well as promoting individual help-seeking behaviour around psychological distress.

With regards to physical presentation of the digital package, it was agreed that the digital package should be informative, free from moving images with the exception of embedded video clips (a ‘diversity and inclusion in the workplace’ consideration), and hosted on a trusted site.

### 3.2. STEP 2: Content Development and Iterative Peer Review

Objectives: To assess the relevance to healthcare workers, the utility, and accessibility of the digital package via a process of peer review.

Methods: The peer review panel consisted of 10 healthcare workers (6 female, 4 male), comprised of 7 medics, 2 registered nurses and 1 paramedic. These individuals self-identified through the professional networks of the project team following a call for peer review to be undertaken within 2 days. They were sent the link to the package and were asked to provide their feedback via an adapted version of the HELM Open RLO-CETL (2005) Evaluation Toolkit for Reusable Learning Objects and Deployment of E-Learning Resources: (https://www.nottingham.ac.uk/helmopen/index.php/pages/view/toolkit). The peer review form contained 10 question items, including consideration of pedagogy, format, usability, navigation, interactivity, delivery, ease of updating, distribution, and access [27]. Reviewers were asked to select a yes/no response for each item, and then expand on their answer if they had further comment. They were first asked whether revision to Version 1.0 was required. Reviewers were then asked whether the focus of the resource was clear and consistent, whether the information was factually correct, and whether the text was well written and in clear sentences. They were asked whether the resources links signposted them to the required information and whether the broad sections of the package were appropriate. Reviewers were then asked to comment on the overall appropriateness of the package with regards layout, images and links. They were asked to comment on the ease or difficulty of initial access to the package via the web link, and whether or not the package could be accessed in different settings (e.g., work or home). Finally, they were asked about the relevance of the package to healthcare professionals.

Replicating the method used in [24] for the purpose of this study, relevance was defined as the appropriateness of content for the specific target audience; utility is defined as how ‘fit for purpose’ the toolkit is with regards how beneficial the content would be to healthcare workers, and how functional the package is for users with regards signposting and locating required information. Accessibility is defined as how easily the package could be used in diverse settings (e.g., hospital, community, home, via mobile device), and how easily the content could be understood by different users.

Following stakeholder feedback and peer review, we produced a ‘minimum viable product (MVP)’ [28], created using Xerte. This is an open-source software for authoring learning objects. Xerte was developed by the University of Nottingham in the UK and is free to download from the Xerte Community website (for more information, see https://www.nottingham.ac.uk/xerte/index.aspx). It is a rapid authoring tool that can be used to create media-rich, interactive and highly accessible content without a requirement for technical or programming knowledge.

Results: Following peer review of Version 1.0, suggestions for change primarily related to further signposting to resources on wellbeing and self-care, together with minor issues of presentation and consistency. All these revisions were made to the package prior to initial distribution of Version 1.0 on 3 April 2020. Further peer review comments made since the initial release of Version 1.0 will be incorporated into an updated version (Version 2.0). The update to create Version 2.0 is planned to take place within approximately 12 weeks of Version 1.0 release. Suggestions to be incorporated into Version 2.0 include minor revisions to the contents page; clarity regarding target audience for sections; defining learning outcomes and pre-requisite technical skills, hardware, web browser and software requirements; inclusion of further signposting material for non-medical workers. Peer reviews also proposed suggestions for future distribution of the package.

Overall, peer reviewers responding to each item rated Version 1.0 as being easy to access (100%, 10/10) and flexible enough for use in different settings (home/work) (100%, 10/10). The package was largely viewed to be appropriate for any healthcare professional (90%, 9/10). The content was perceived to be factually correct (100%, 10/10), with a clear and consistent focus (90%, 9/10). The broad sections were seen to be appropriate (100%, 10/10), with clear, well-written text (90%, 10/10). With regards presentation and signposting, reviewers perceived the package to be appropriately presented with regards images, layout and links (90%, 9/10), and the resource links were seen to provide the information needed (90%, 9/10).

### 3.3. STEP 3: Delivery and Evaluation

Objectives: To estimate initial interest in the package through engagement within 7 days of release, and to determine intervention fidelity through quantitative assessment of user experience, content relevance, utility and accessibility.

Methods: We replicated procedures and success criteria described elsewhere for the evaluation of digital packages [24]. Healthcare workers and healthcare students were recruited over 3 days via professional networks and provided with a link to Version 1.0 of the digital package. Instructions were simply to review the tool and provide feedback using a standard evaluation form with 20 question items to assess the fidelity and implementation qualities of the package. The evaluation form was developed by two members of the research team and the question items were peer reviewed by 2 healthcare workers prior to use. Respondents were required to classify their occupation: healthcare professional, healthcare academic, other key worker, or healthcare student. Participants were asked to respond (yes/no) whether they had been able to access the full functioning package via the web link; whether they had understood the information and gained sufficient knowledge from it; whether they had practically used the information at work or at home (and specify how) and, if not, whether they perceived the information to have future value in this regard. They were asked to indicate which of the package sub-sections they viewed, by tick box response. Participants were asked whether the resource was applicable to any healthcare professional (yes/no, then rate the relevance to healthcare workers on a scale of 1–10), and whether using the resource was time well spent (yes/no). They were required to rate the level of perceived burden to complete the package (on a scale of 1–10). They were asked to respond (yes/no) about any technical challenges they experienced while using the package, with regards their own skills, and also technical issues with the platform; and whether the cost burden was acceptable to them (the package was free to use but naturally incurred a personal cost—burden of time—to complete it). Participants were asked to rate on a scale of 1–10 how they felt about this package being available to all healthcare workers; whether they felt the content was useful and whether the resource was easy to navigate and use. Finally, they were asked whether they would recommend the package to a colleague (yes/no), and whether they had any suggestions for improvement should updates be made in the future.

We did not impose any time restrictions for package completion or specify the order in which materials should be viewed. The evaluation was capped at 55 participants to expedite the evaluation process in order for the package to be timely enough to support healthcare workers during the COVID-19 pandemic.

The evaluation form containing the measures of fidelity and implementation qualities described above was sent to participants by email alongside the link to the package, and non-responders were followed up 2 days later. Data were collected by email return of the evaluation form (*n* = 14), or completion of the form via structured telephone interview (*n* = 41) with a member of the research team.

(a) Assessment of Fidelity (Delivery and Engagement)

Constructs of fidelity were assessed that measured the extent to which the intervention was delivered in line with the protocol (‘fidelity of delivery’) and that content was engaged with by participants (‘fidelity of engagement’). Fidelity of delivery included (i) assessment of the dose delivery of intervention components as per protocol (receipt of functioning link to the digital package yes/no), and (ii) the actual dose received (access to each section expressed as % completion rate). Success was pre-defined as >90% for per-protocol delivery, and >75% digital package completion (expressed as the % of full content accessed).

Fidelity of engagement with intervention content was measured through 4 self-reported dichotomous question items assessing (i) whether participants understood the package content (yes/no), (ii) whether they gained sufficient knowledge provided by the digital package (‘intervention receipt’) (yes/no), (iii) whether they used this knowledge in skills in daily working life (‘intervention enactment’) (yes/no, with open-ended response as to how), and (iv) whether they perceived that they might use this knowledge in the future (yes/no). Success was pre-defined as >90% for items (i) and (ii), and >30% for item (iii) (given the exceptionally short time frame from digital package use to fidelity assessment), and >50% for item (iv).

(b) Assessment of Implementation Qualities

Participants were asked to report on practicality, resource challenges, attitudes towards the digital package, acceptability, usability and cost.

Practicality was defined as the usability of the package despite limited resources. Items included one dichotomous and one 1–10 scale rating, assessing (i) whether the digital package could be used by any healthcare professional (yes/no), and (ii) level of burden (1 = zero burden, 10 = highest burden). Success was pre-defined as >75% yes response for (i), and average score of <6 for (ii). Resources challenges were defined as (i) time challenges (yes/no), (ii) technical challenges, defined as lack of required technical skills (yes/no), (iii) financial challenges (yes/no) or other (free text). Success was pre-defined as <25%, reporting one or more resource challenges. Attitudes were defined as positive views towards the digital package and assessed by a 1–10 rating scale: how did you feel about the availability of this package (1 = very negative, 10 = highly positive). Success was pre-defined as average score of >6. Acceptability was defined as whether the measure is appropriate for those who will use it. This included two dichotomous items with open-ended explanation, and one 1–10 scale response: (i) whether the information contained in the digital package was appropriate for their needs (yes/no), (ii) whether it contained meaningful information (yes/no) and, (iii) the perceived usefulness of the package (1 = not at all useful, 10 = extremely useful). Success was pre-defined as >75% for (i) and (ii), and an average score of >6 for (iii).

Usability was defined as whether the package was perceived to be easy to use. This was assessed by one 1–10 scale item and one dichotomous item measuring: (i) ease of navigation (1 = not at all easy, 10 = extremely easy) and (ii) whether they had experienced any technical difficulties, defined as technical problems, with the package functioning (yes/no). Success was pre-defined as an average score of >6 for (i), and <25% reporting a technical difficulty for (ii).

Since this digital package is freely available online, cost was defined here as the perceived human cost implications for healthcare workers to take time out to complete the resource, completed via a dichotomous item (acceptable cost implications/unacceptable cost implications). Success is defined as >75% reporting acceptable cost implications.

Results: We developed a digital package called: ‘Psychological Wellbeing in Healthcare Workers: Mitigating the Impacts of COVID-19’ [29]. Version 1.0 (last updated on 2 April 2020) is comprised of 88 slides within six sections (see Table 1) and is available at: https://www.nottingham.ac.uk/toolkits/play_22794 (see Supplementary Materials).

The Template for Intervention Description and Replication (TIDieR) checklist and guide was used to inform the description of the package [30]. Use of the package requires no prior knowledge or training, and the mode of delivery is via web link, with the intention that the resource would be utilised independently and individually by healthcare workers (or healthcare students and academics) at a time and location of their choosing. To complete the entire digital learning package (including access to all additional resources signposted from within the package), it takes approximately 120 min. As proposed by stakeholders, the package is designed for flexible access, with ‘dip-in and dip-out’ learning or signposting, and access to each section is not dependent upon completion of prior sections. Information is relevant to all healthcare workers as well as healthcare academics and healthcare students. Therefore, it is generic and not personalised or tailored, although users can choose which elements to engage with, how and when they are accessed. This approach also allows team leaders to signpost particular sections to their teams to support their existing provisions for psychological wellbeing. The intervention is designed so that content and links can be periodically checked and updated by the authors in order to generate subsequent versions and ensure that content remains in line with current policy and practice.

The package was accessed 17,633 times and had >50,000 exposures via social media within 7 days of release. Results of the fidelity assessment are shown in Table 3. There were 55 participants (49 employees, 6 students) completing the evaluation. Participants included medical doctors (*n* = 9; secondary care *n* = 8, primary care *n* = 1), nurses (*n* = 22; secondary care *n* = 16; primary care/community *n* = 2, student *n* = 4), midwives (*n* = 5; registered *n* = 3, student *n* = 2), dentist (*n* = 1), psychological professions (*n* = 3), allied health professionals (*n* = 9; physiotherapists *n* = 3, occupational therapist *n* = 1, speech and language therapist, *n* = 1 dietician *n* = 1, radiographer *n* = 1, orthotist *n* = 1, healthcare assistant *n* = 1), paramedics (*n* = 4), pharmacist (*n* = 1), and wider healthcare workers (*n* = 5; human resource advisor *n* = 1, health informatics officer (*n* = 1), laboratory technician *n* = 1, domestic assistant *n* = 1, porter *n* = 1).

All of the pre-defined success criteria were met for the fidelity assessment (both delivery and engagement), and implementation qualities (practicality, resource challenges, attitudes, acceptability and usability). Within just 7 days of package release, 82% of healthcare participants reported having used the information provided in their work or home lives, and 100% anticipated they would use it in the future.

Many healthcare workers reported that following engagement with the package, they had already taken further actions (‘intervention enactment’) to emotionally support colleagues and family members, considered training in psychological first aid (PFA), called a telephone helpline, or engaged with advice around coping with emotions.

Many had accessed the interactive elements (e.g., video clips) and used apps signposted from within the package. They reported sharing the information in the following ways: circulating the package link around their clinical teams, colleagues and students; sharing the resource with external professional networks via email, print media, websites and social media; including a link to the digital package within their organisation’s COVID-19 Staff Health and Wellbeing provisions; uploading the package to internal educational resource portals; printing posters and guidance documents (that were signposted from within the package) and placing them in shared areas such as staffrooms or noticeboards.

## 4. Discussion

This study reports on the rapid development and evaluation process for an e-package to support the psychological wellbeing of healthcare workers during and after the COVID-19 pandemic. The package was developed using Agile methodology which included rigorous, iterative peer review processes and an evaluation with a diverse group of healthcare workers from the UK. This work has fully described the development processes, and confirmed the fidelity of package delivery and engagement, as well as the package implementation qualities, using processes that have been used successfully elsewhere [24]. The end result is an online support package that can immediately be provided to healthcare workers in hospital or community settings.

It is notable that this package was very highly accessed within just 7 days of release. This demonstrates an exceptional level of interest in this package as a mechanism for supporting psychological wellbeing, not least because the distribution and dissemination plans have not yet been developed. While the package was developed with a UK audience in mind, much of the content and advice contained within it has relevance to an international audience, with the exception of some of the materials in the further resources section that are UK specific (e.g., telephone helplines). The wider applicability of the package has been confirmed by the extent of initial exposure on social media and re-distribution of the package by healthcare organisations and professional bodies within the first 7 days to include an international audience (e.g., UK, USA, Europe and China to date).

Since this project was a rapid response to COVID-19, with a need for immediate package implementation, the evaluation was limited to a small sample of healthcare workers from the UK. There is scope for further evaluation studies to investigate healthcare workers’ perceptions towards and use of the package and any resulting changes in actions (e.g., communication, team approaches, self-care and managing emotions). This could be examined in any occupational groups but particularly frontline healthcare workers with greatest exposure to COVID-19, such as emergency personnel. Since COVID-19 information and support is rapidly evolving, the package could be updated in due course, and there is scope to adapt the resources within the content and test the package in international contexts.

## 5. Conclusions

The COVID-19 pandemic presents significant challenges for healthcare services. We have established a need to develop a digital support package around psychological wellbeing in healthcare through stakeholder consultations. We have met this need through the rapid development of an evidence-based digital package on psychological wellbeing for healthcare workers, which is relevant to all healthcare workers in the UK as well as healthcare academics and students. Evaluation demonstrated that the package has high fidelity with regards delivery to, and engagement of, healthcare workers. Assessment of implementation qualities showed high usability and practicality, with low perceived burden for completion and acceptable cost implications. This digital package is considered to be appropriate for any UK healthcare professional as well as healthcare academics and students, with much of the content having international relevance. Overall, the content was perceived to be useful, meaningful and appropriate to the needs of healthcare workers. We recommend that this package is distributed to all healthcare workers to supplement strategic health and wellbeing provisions for employees during and after the COVID-19 pandemic.

## Figures and Tables

**Table 1:** Outline of Package Content for Version 1.0.

Section	Content (Version 1.0, Last Updated 02.04.2020)
Quick Links	Links to relevant areas of the learning tool
Psychological Impacts	Specific Threats to Psychosocial Wellbeing from COVID-19
	Healthcare Workers and First Responders
	Healthcare Groups Most at Risk
	Remember… (Normalising Psychological Responses)
	Key Symptoms of Sustained Stress
	Risk Factors for Psychological Ill-Health
	Mitigating the Risk (Training and Preparation)
Psychologically Supportive Teams	The Impact of Workplace on Psychological Wellbeing
	Building Resilience in Your Teams
	Create a Psychologically Safe Space in the Workplace
	Key Actions for Team Leaders and Managers
	How to Improve the Working Environment
	Working under Pressure in a Team
	Section Summary
Communication	Sourcing and Providing Information
	Communication Approaches
	Clarity Reduces Stress: Planning and Roles
	Clarity Reduces Stress: Guidelines and Resources
	Language Matters
	Addressing Social Stigma
	Being Informed or Being Overwhelmed?
	How to Talk to Children about Coronavirus
	Helping Children Cope with Stress
	Advice for Young People with Anxiety
Social Support	Accessing Support in the Workplace
	Peer Support and the Going Home Checklist
	Accessing Family and Community Support
	Supporting and Signposting Others: Psychological First Aid
	Remote Psychological Support Options
Self-Care	Rest and Work Breaks
	Managing Fatigue
	Importance of Sleep
	Sleep Improvement
	Coping with Isolation and Confinement
Manage Emotions	Making Morally Challenging Decisions
	Choosing Between Difficult Options
	Moral Injury or Psychological Growth?
	Coping in Demanding Environments—Challenge or Threat?
	How to Manage Feelings of Guilt
	Coping with Grief and Death
	Managing Stress, Anxiety and Low Mood
	Resources for Mental Wellbeing in Healthcare Staff
	Mindfulness (and Mindfulness Resources)
	Signs of Burnout
	COVID-19 Resilience Tips from a Front-Line ICU Nurse
	Signs of Post-Traumatic Stress Disorder (PTSD)
	When Psychological Impacts Require Treatment
	Tips for Managing Emotions
	Tips on Managing Anxiety
Further Resources	Telephone Helplines
	British Psychological Society: COVID-19 Guidance
	Caring for Doctors Caring for Patients
	HAWN Training Package—for HCAs, Nurses and Midwives
	Support the Workers—Briefing Notes
	Downloadable Wellbeing Posters
	World Health Organisation (WHO)—Mental Health Guidance
	Public Health England—Mental Health Guidance
	MIND—Mental Health Guidance
	RCPCH—Wellbeing and Resilience Guidance
	Stress and Resilience at Work
	Royal College of Psychiatrists—Mental Health Guidance
	Academy of Medical Royal Colleges—Directory of Support
Developers	This e-resource has been compiled by…

**Table 2:** Mapping Research Questions (RQ) to Digital Package [26].

Research Questions (RQ)	Digital Package
Is there a clear health need which this package is intended to address?	Psychological wellbeing in healthcare workers.
Is there a defined population who could benefit from this toolkit?	*Directly*: Healthcare workers (including, but not limited to, nurses, doctors, allied health professionals); healthcare academics; healthcare students.
	*Indirectly*: Patients and the public, through protecting the psychological wellbeing of the healthcare workforce.
Is the package likely to reach this population and, if so, is the population likely to use it?	The package is open access and so reach and uptake data cannot be accurately specified due to the nature of rapid circulation in response to COVID-19. However, reach of the package via one platform only (Twitter impressions and engagement) and confirmed uptake (individual response) will be reported within 7 days of package release (e.g., minimum reach). We have included descriptions of mechanisms for sharing and impact of materials provided by users in this study.
Acceptability and usability	Determined by peer reviews, and package usability evaluation questions.
Demand	Confirmed by consultations with healthcare workers.
Implementation	High fidelity: toolkit has been tested ‘in the wild’ (with competing demands on the user’s attention).
Practicability	Xerte online package requires no technical skills or login and is accessible across a range of commonly used operating systems and devices.
Adaptation	Package can be reviewed and updated without compromising fidelity/integrity.
Integration	Publicly accessible, hosted on a trusted site, integrated into an existing repository of e-learning resources.
Is there a credible causal explanation for the package to achieve the desired impact?	Credibility of authors and sources (e.g., subject experts, professional bodies, government/WHO reports). Package was developed through multi-professional consultation.
	Content addresses knowledge gaps and needs as identified in stakeholder consultation.
	Dual purpose:
	[a] As an educational tool on psychological wellbeing in healthcare (e.g., for healthcare students), and
	[b] Provided as part of a wider package of psychological support for healthcare workers during/after the COVID-19 pandemic.
	No human support element is required to deliver the digital package.
What are the key components of the package? Which ones impact on the predicted outcome, and how do they interact with each other?	Key components:
	Requires ~2 h per user to complete full package, although this is variable since individual sections can be viewed separately.
	Free access to all users.
	Content is not individually tailored, although context or discipline-specific information can be provided alongside.
	Section completion does not rely on completion of earlier sections.
	Package is timely in response to COVID-19 (to maximise user compliance).
	Format is a simple interactive e-learning package to maximise implementation and scalability.
	Content and signposting to further resources (Table 1).
What strategies should be used to support tailoring the package to participants over time?	Full package completion is intended. However, there is opportunity for tailoring, adaptive learning and user choice. Users may self-select components of interest, e.g., to individually tailor order and dosage of learning, as well as access to external signposted resources. Context-specific information (e.g., job-related, organisation type) or discipline-specific information (e.g., nursing, medicine, allied health) can be included separately.
What is the likely direction and magnitude of the effect of the package or its components compared to a comparator that is meaningful for the stage of the research process?	Demonstrated benefit to healthcare workers, package shown to be acceptable and feasible.
	Toolkit will remain stable over the medium term (although periodical updates will be required due to the nature of a pandemic and the potential for changing advice).
	Immediate reach and uptake will be determined by package views and Twitter reach within 7 days of release.
	Direction and magnitude of effect to be tested in future research.
Has the possibility of harm been adequately considered? And the likelihood of risks or adverse outcomes assessed?	Provision of accurate information and advice relating to psychological wellbeing—includes advice from medical doctors, psychologists, and other health professionals as well as official guidance from relevant societies and health services.
	Stakeholder consultation suggested low risk of content misinterpretation.
	Potential for package to encourage more healthcare providers to offer employee health and wellbeing provisions—this could result in identification of psychological distress in their employees. However, package contains guidance on actions by managers to create psychologically supportive environments.
	No issues with data security or privacy breaches, no personal data collected.
	No adverse outcomes were reported during evaluation testing.
	Free package means there are no opportunity costs for employers.
Has cost been adequately considered and measured?	Free and widely accessible delivery platform (Xerte online package).
	Long-term maintenance/updating costs would need to be calculated in a formal health economic analysis if the package were to be tested in a full-scale trial.
	Estimated 5 h per year maintenance for toolkit authors.
What is the overall assessment of the utility of this intervention? And how confident are we in this overall assessment?	High overall utility of the package—based on its potential to increase knowledge on psychological wellbeing in healthcare workers in diverse professions, as well as academic environments.
	Content development involved stakeholder consultation.
	Based on reach estimates from one working day, this has potential for wide reach and high uptake, with low development and maintenance costs. It is immediately scalable, has no reported adverse effects, and has positive evaluation from healthcare workers from diverse specialties.
	True assessment of confidence requires testing in a future trial. However, the developed toolkit could easily be incorporated into routine organisational practice in its current form.

**Table 3:** Intervention Fidelity and Implementation Testing.

Assessment Type (*n* = 55)	*n*	Actual Success Rate *n* (%) or Mean (SD)	Pre-Defined Success Rate *n* (%) or Mean
**eFidelity Assessment**			
*Fidelity of Delivery*			
Per-protocol delivery (functioning link)	55	55 (100)	>90% *
Toolkit completion rate:			
Main sections	55	55 (100)	>75% *
Further resources	55	49 (89)	
*Fidelity of Engagement*			
Understanding of the toolkit	55	55 (100)	>90% *
Intervention receipt (perceived knowledge)	55	55 (100)	>90% *
Intervention enactment (knowledge use, 1 w ^†^)	55	45 (82)	>30% *
Perceived enactment (future use)	55	55 (100)	>50% *
**Implementation Qualities**			
*Practicality*			
Use by any healthcare professional	55	53 (96)	>75% *
Relevance to any healthcare professional	55	9.51 (0.79)	>6 *
Level of burden	55	2.56 (1.81)	<6 *
*Resource Challenges*			
Time challenges	54	0 (0)	<25% *
Technical challenges (skills)	54	0 (0)	<25% *
Financial challenges	54	0 (0)	<25% *
*Attitudes*			
Perceptions toward availability	55	9.78 (0.74)	>6 *
Would recommend to others	55	55 (100)	>75% *
*Acceptability*			
Appropriate for needs	54	54 (100)	>75% *
Contains meaningful information	55	55 (100)	>75% *
Perceived usefulness of the toolkit	55	9.47 (0.96)	>6 *
*Usability*			
Ease of navigation	55	9.76 (0.67)	>6 *
Technical difficulties (functioning)	55	0 (100)	<25% *
*Cost*			
Acceptable cost implications	54	54 (100)	>75% *

^†^ 1 week after package release. * meets pre-defined success rate.

## Supplementary Materials

The digital package is available online at https://www.nottingham.ac.uk/toolkits/play_22794.
